# Melatonin: a modulator in metabolic rewiring in T-cell malignancies

**DOI:** 10.3389/fonc.2023.1248339

**Published:** 2024-01-08

**Authors:** Seema Rai, Gunja Roy, Younis Ahmad Hajam

**Affiliations:** ^1^ Department of Zoology Guru Ghasidas Vishwavidyalaya, Bilaspur, India; ^2^ Department of Life Sciences and Allied Health Sciences, Sant Bhag Singh University, Jalandhar, India

**Keywords:** T-cell malignancy, melatonin, metabolic rewiring, tumor microenvironment, T-cell exhaustion, cancer

## Abstract

Melatonin, (N-acetyl-5-methoxytryptamine) an indoleamine exerts multifaced effects and regulates numerous cellular pathways and molecular targets associated with circadian rhythm, immune modulation, and seasonal reproduction including metabolic rewiring during T cell malignancy. T-cell malignancies encompass a group of hematological cancers characterized by the uncontrolled growth and proliferation of malignant T-cells. These cancer cells exhibit a distinct metabolic adaptation, a hallmark of cancer in general, as they rewire their metabolic pathways to meet the heightened energy requirements and biosynthesis necessary for malignancies is the Warburg effect, characterized by a shift towards glycolysis, even when oxygen is available. In addition, T-cell malignancies cause metabolic shift by inhibiting the enzyme pyruvate Dehydrogenase Kinase (PDK) which in turn results in increased acetyl CoA enzyme production and cellular glycolytic activity. Further, melatonin plays a modulatory role in the expression of essential transporters (Glut1, Glut2) responsible for nutrient uptake and metabolic rewiring, such as glucose and amino acid transporters in T-cells. This modulation significantly impacts the metabolic profile of T-cells, consequently affecting their differentiation. Furthermore, melatonin has been found to regulate the expression of critical signaling molecules involved in T-cell activations, such as CD38, and CD69. These molecules are integral to T-cell adhesion, signaling, and activation. This review aims to provide insights into the mechanism of melatonin’s anticancer properties concerning metabolic rewiring during T-cell malignancy. The present review encompasses the involvement of oncogenic factors, the tumor microenvironment and metabolic alteration, hallmarks, metabolic reprogramming, and the anti-oncogenic/oncostatic impact of melatonin on various cancer cells.

## 1 Introduction

The human body’s homeostasis depends on the blood and immune system derived from the hematopoietic stem cells (HSCs), which are multipotent stem cells that can generate various types of blood cells. HSCs produce erythrocytes, which transport life-sustaining oxygen; Thrombocytes, which help with blood clotting; and leukocytes primarily white blood cells, which fight infections and disease. These are derived from versatile sources of stem cells. HSCs coordinate a key transition within this milieu of white blood cells and produce T lymphocytes also known as T-cells, which act as guardians of the immune system (1).

T cells play a crucial role in immune response by generating various cytokines and interleukins, while B cells produce antibodies. These cell types exhibit different cell surface characteristics, functions, and developmental pathways. Cell surface markers are protein molecules expressed on the surface of a cell that particular antibodies may recognize. They may be used as an indicator for cell identification, functions, and conditions. Neoplastic cells are those that have grown abnormally and uncontrollably, resulting in the creation of tumors or malignancies. By analyzing the cell surface markers on neoplastic cells, their origin (which type of normal cell they are derived from), nature (whether they are benign or malignant, and what subtype of cancer they belong to), and involvement in the development of the lymphoproliferative disease can be recognized (2). Dysregulation in the functioning of immune cells can lead to serious disorders, and immune dysfunction may contribute to the development and progression of malignancies or cancer (3). Cancer cells are aberrant cells that have undergone abnormal development and uncontrolled proliferation. Different cells of the immune system, including T cells and natural killer (NK) cells, play a crucial role in shriveling the growth and proliferation of cancer cells, as well as eliminating malignant cells. Therefore, targeting NK cells and T-cell immunotherapy hold significant promise as potential tools for cancer treatment (4). Several factors, such as genetic predisposition (5), human T-cell leukemia virus (HTLV-1), Epstein-Barr virus, and autoimmune disorders, have been implicated in the pathogenesis of T-cell malignancies (6).

T-cell malignancies are a diverse set of illnesses that each result from the clonal evolution of damaged T cells at different phases of life. Nonetheless, acute lymphoblastic leukemia (T-ALL), the most frequent kind of T cell malignancy found in children has been reported to account for 15% to 25% (7). A non-Hodgkin lymphoma with a biology comparable to T-ALL is T-lymphoblastic lymphoma (T-LLy). The human T cell lymphocytic virus type 1 (HTLV1) is the primary cause of the exceedingly aggressive blood malignancy known as adult T cell leukemia/lymphoma (ATLL) (8, 9).T cell large granular lymphocytic leukemia (T-LGL) and T prolymphocytic leukemia (T-PLL) are two further uncommon types of T cell leukemia (10). The two main types of T cell lymphomas are cutaneous T cell lymphoma (CTCL) and peripheral T cell lymphoma (PTCL). The two most prevalent subtypes of CTCL are mycosis fungoids (MF) and Sezary syndrome (SS), which together account for the majority of cases. Angioimmunoblastic T cell lymphoma (AITL), extranodal natural killer (NK)-T cell lymphoma (ENKTL), enteropathy-associated T cell lymphoma (EATL), hepatosplenic T cell lymphoma (HSTCL), and PTCL-not otherwise specified (PTCL-NOS), the most prevalent of the group, are among the subtypes of PTCL that can be distinguished (11). According to previous reports, the incidence of cancer is expected to increase significantly by 2050, with about 19.3 million new cases annually, making it the second leading cause of death worldwide (12). The report by the World Health Organization (WHO) confirms that as of 2018, there were 9.6 million cancer-related deaths worldwide, with morbidity and mortality rates increasing significantly over time. Cancer has become a major health issue globally, and it requires immediate attention from researchers and clinicians. The Global Cancer Observatory (GLOBOCAN) of the International Agency for Research on Cancer of the World Health Organisation predicts that by 2040, there will be 27.5 million new cases of various cancers annually. This represents an increase of 61.7% when compared to recent statistics (13).

Chemotherapy, radiation therapy, and surgery represent the conventional approaches recommended and employed by doctors for treating cancer patients. Regrettably, these methods often come with a host of adverse effects and provide no guarantee against cancer recurrence. Consequently, there exists a pressing demand for novel therapeutic agents capable of enhancing post-treatment survival and quality of life for cancer patients. These innovative agents might take the form of biological molecules designed to modulate the immune system and possess anti-cancer properties. One illustrative instance of such a biological molecule is melatonin, a naturally occurring hormone renowned for its relatively minimal side effects when compared to synthetic drugs. Melatonin also exhibits the ability to target specific genes and DNA associated with the development and progression of cancer. In recent years, numerous studies have been conducted to delve into the multifaceted functions and role of melatonin in human health and various disease states (14).

**Figure 1:**
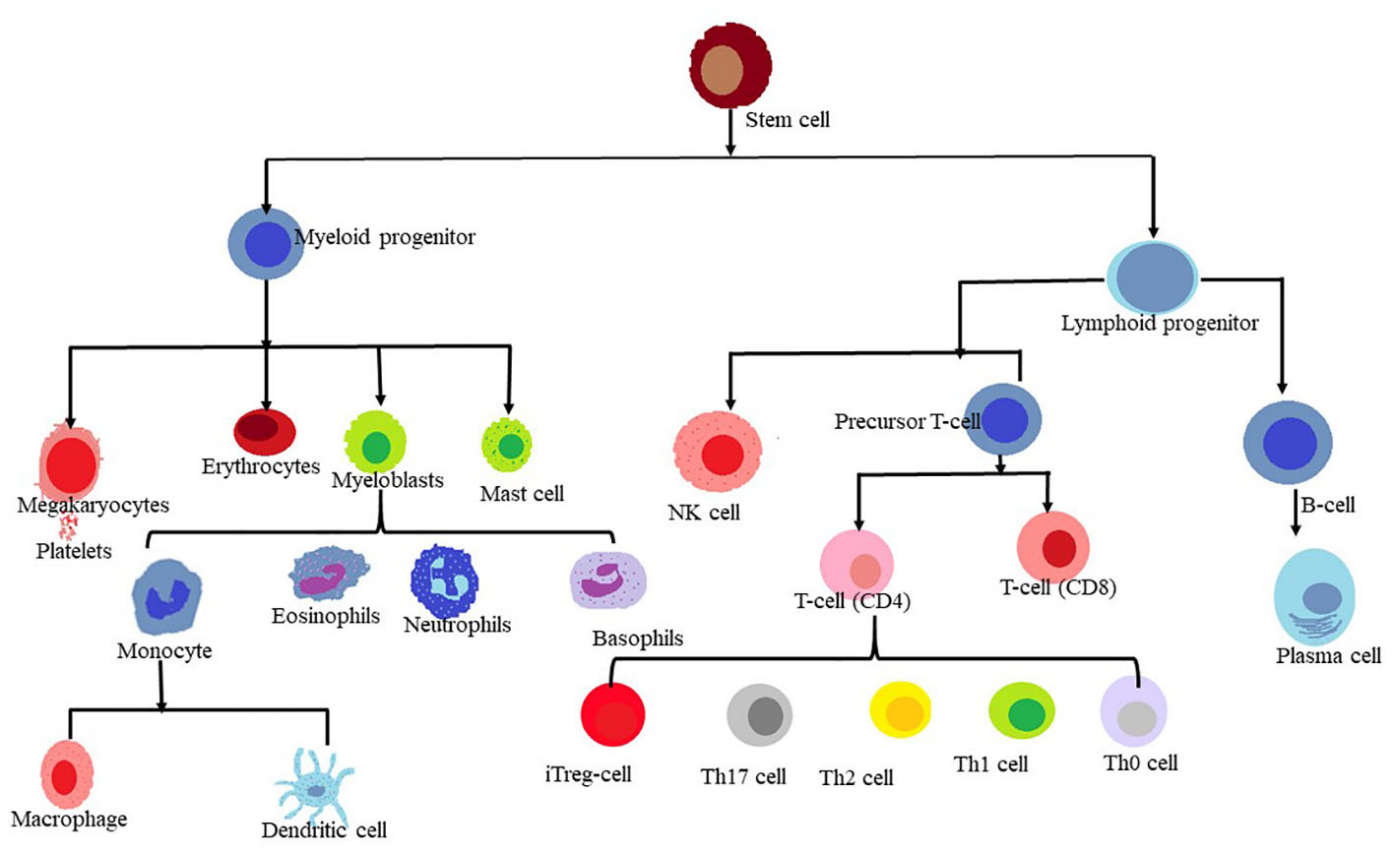
Lineage of myeloid and lymphoid cells.

In recent years, extensive study has been conducted to discover the various functions, uses, and properties of melatonin in human physiology and pathology. Melatonin is an indolamine and neurohormone secreted rhythmically by the pineal gland. Aaron B Lerner in 1958, isolated melatonin from the bovine pineal extract and identified it chemically as 5-Methoxy-N-Acetyl tryptamine. The molecular mechanism for the physiological effects of melatonin has been explained which provides evidence regarding its involvement in the regulation of the sleep-wake cycle, circadian rhythms, reproduction, immunological response, apoptosis, proliferation, metastasis, angiogenesis, and oxidative stress via receptor-mediated (Nuclear and membrane receptors) and nonreceptor activities carried out through non-receptor-dependent pathways (15–20). There are numerous cytoplasmic and mitochondrial binding sites for melatonin in addition to membrane and nuclear receptors (15, 21–28). It is a lipophilic molecule and hence virtue makes it a potent antioxidative agent, thereby it reduces the free radicals like- reactive oxygen species (ROS) and reactive nitrogen species (RNS) generation and maintains a balance between the anti-oxidant and ROS/RNS (14) (**Figure 1**).

In addition, melatonin is a well-known molecule that can rewire metabolic pathways. Melatonin has also been found to have a significant role in the regulation of various types of cancer. It exhibits anti-angiogenic and anti-metastatic properties and has the potential to prevent cell necrosis, as observed in some studies on breast cancer cell lines. Moreover, melatonin regulates transcription factors such as NF-kB and influences the production of cytokines and interleukins (29, 30). A recent study using a nude mice model demonstrated that melatonin treatment (10 mg/kg) via intraperitoneal administration for three weeks resulted in a reduction in tumor size (31). It regulates factors such as vascular endothelial growth factor (VEGF), hypoxia-inducible factor-1α (HIF-1α), and human epidermal growth factor receptor 2 (HER-2) (32). In patients with lung cancer caused by smoking cigarettes, a significant increase in the levels of pro-inflammatory cytokines, which are signaling molecules involved in promoting inflammation, has been observed (32). However, it has been studied that the administration of melatonin can counteract these effects by reducing neutrophil counts and inhibiting the production of pro-inflammatory cytokines such as tumor necrosis factor-alpha (TNF-α), interleukin-1beta (IL-1β), interleukin-8 (IL-8), and interleukin-6 (IL-6) (33).

Numerous investigations also supported the anticancer and oncostatic properties of melatonin, which are mediated by various modes of action (34, 35). Melatonin was further employed in conjunction with other anticancer therapies to supplement standard treatments and lessen negative effects (34–36).

## 2 Cancer and oncogenic factors

**Figure 2:**
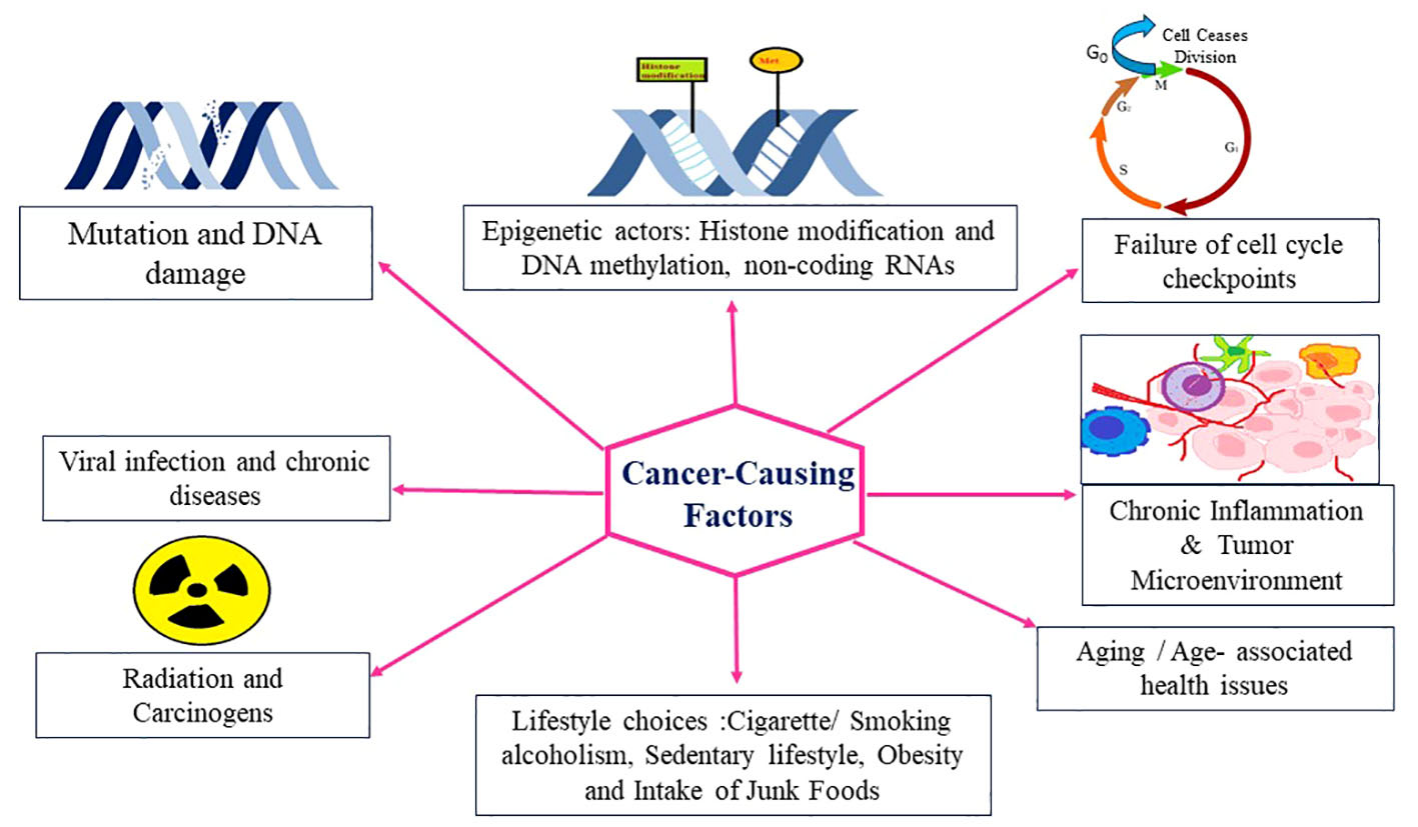
Different factors responsible for cancer.

Cancer is a multifaceted disease characterized by uncontrolled cell growth and proliferation, leading to the formation of malignant tumors. It arises from a combination of genetic and environmental factors, which interact to initiate and promote oncogenesis. Understanding the intricate interplay of these oncogenic factors is crucial for elucidating the underlying mechanisms of cancer development and identifying potential targets for therapeutic interventions. In recent years, significant advancements in cancer research have shed light on the diverse array of oncogenic factors involved in this complex disease. Genetic alterations play a fundamental role in the development of cancer (34). Mutations in key genes, such as oncogenes and tumor suppressor genes, can disrupt the delicate balance of cellular processes, leading to uncontrolled cell growth. Oncogenes are genes that, when mutated or overexpressed, promote cell proliferation and survival, while tumor suppressor genes act as guardians of the genome, regulating cell cycle progression and preventing the formation of tumors. Research has uncovered numerous oncogenes and tumor suppressor genes, including well-known examples like TP53, KRAS, and BRCA1/2, which have provided critical insights into the molecular basis of cancer. In addition to genetic alterations, Carcinogens present in the environment, such as tobacco smoke, ultraviolet radiation, and certain chemicals, can induce DNA damage and initiate oncogenic processes, and lifestyle factors, such as diet, physical activity, and exposure to toxins, can influence cancer risk by modulating various signaling pathways and epigenetic modifications contribute significantly to cancer development (**Figure 2**).

### 2.1 Tumor microenvironment and metabolic alteration in malignant cells

Cells require macromolecules that support them in growth and division similarly malignant cells also require macromolecules for their development and these macromolecules are synthesized from scratch via rewiring the metabolic pathways inside the cells or may be obtained from other sources, to meet the metabolic demands required for their development and proliferation (37). The demand for malignant cells is aided by the tumor microenvironment (TEM). This is a complex dynamic network of host components that are recruited and reprogrammed by the tumor to support its proliferation and survival. The TEM is composed of various cellular and noon-cellular elements that interact with each other and with the malignant cells through physical interaction and molecular signals. The cellular components comprise immune cells –macrophage, dendritic cell, T-cells, B-cells, NK-cells, and mast cells, fibroblasts these are the main source of extracellular matrix (EMC) proteins and growth factors; endothelial cells, which form blood vessels that supply oxygen nutrients to the growing tumors and other cell types such as adipocytes, pericytes, and neutral cells (38, 39). The TEM plays a crucial role in the development, proliferation, metastasis, and response to therapy of tumors by affecting the biological nature and phenotype of tumor cells. The TEM can promote tumor cell proliferation, survival, migration, angiogenesis, stemness, epithelial-mesenchymal transition (EMT), drug resistance, and immune evasion. However, the TEM is not a static or homogenous entity, but rather a dynamic and heterogenous that modulates itself over time. And these modifications also alter the metabolic reprogramming in tumor cells (38, 40, 41). Previously it was believed that the TEM are consequence of the metabolic by-products of tumor cells, such as lactate, ammonia, and ROS. However, studies have shown that TEM can also initiate and sustain the metabolic rewiring of tumor cells. These tumor cells show some hallmarks in terms of metabolic reprogramming which favors their survival, development, and proliferation (41, 42).

The hallmarks of cancer cell metabolic rewiring involve aerobic glycolysis (Warburg effect), a hypoxic condition, that contributes to the cancer cell’s transformation by providing a tumor microenvironment (TEM) (43). Aerobic glycolysis or Warburg effect is the phenomenon of glucose fermentation into lactate or lactic acid in the presence of oxygen i.e., in aerobic conditions. This process provides anabolic carbons that can be used in biosynthetic pathways. Glucose uptake in normal cells is strictly regulated but, malignant cells have been reprogrammed to fuel unconstrained proliferation (44) and many malignant cell types have been reported to take up glucose at unusually elevated rates. Glucose could be transformed into a variety of cell-building components like lipids, amino acids, and, nucleotides, necessary for the development and proliferation of malignant cells. Aerobic glycolysis promotes the generation of electron carriers, which are required as cofactors in the redox process in cells. Elevated glucose uptake allows for increased synthesis of the reducing equivalent NADPH via the oxidative branch of the pentose phosphate pathways, whereas fermentation entails regeneration of the oxidizing NAD+ via the activity of lactate dehydrogenase (LDH) (45). These electron carriers are required for cellular redox balance and biosynthesis. Under hypoxic circumstances, aerobic glycolysis optimizes ATP generation (45). Hypoxia, or low oxygen level, is prevalent in solid tumors due to insufficient blood flow. Hypoxia can activate hypoxia-inducible factor-1α (HIF-1α), a transcription factor that controls numerous glycolysis-related genes. HIF-1α is a key regulator of cellular response to hypoxia condition and it also regulates the expression of genes that are responsible for angiogenesis, erythropoiesis, and acidosis regulation (45). HIF-1α induces genes like vascular endothelial growth factor (VEGF) (46). Angiogenesis generation of new blood vessels in developing malignant cells in an uncontrolled manner to meet the demand for oxygen and nutrients. On the contrary, it results in some disorganized structure with branching and splitting forming a chaotic and leaky network (47). Such a clumsy structure leads to inadequate blood flow, anaerobic conditions (hypoxic environment), and the generation of free radicals. Aerobic glycolysis leads to the generation of lactate which makes the tumor environment acidic and increases the free radical load. “Earlier” lactate is considered a by-product of enhanced aerobic glycolysis and accumulates in malignant cells, raising the pH of the tumor microenvironment but a recent investigation by Brandon et al. suggests that it fuels the tricarboxylic acid cycle (TCA) (48). Lactate promotes HIF-1α activity by blocking the prolyl hydroxylases 2 (PDH 2). However, the specific mechanism is still unclear (49). When PDH 2 gets inhibited HIF-1α localized into the nucleus and binds with its other subunit HIF-1β. Further, this complex binds with hypoxia-responsive elements (HREs) and activates the gene expression for the *NOX* genes (*NOX1, NOX 2, NOX 4*, and *NOX 5*) NOX enzymes also contribute to the production of free radicals Superoxide and hydrogen peroxide (50) by transferring the electrons from NADPH to oxygen (51). All these factors contribute to the establishment of a suitable condition for the development and proliferation of malignant cells known as a tumor microenvironment (TEM). TEM favors the abnormal development and proliferation of malignant cells and is associated with various pathways such as the PI3K/Akt/mTOR pathways, insulin-like growth factor-1 (IGF-1), hypoxia-inducible factor-1 (HIF-1), and NF-KB. These pathways play a direct role in promoting tumor development in the tumor microenvironment (TME), a condition characterized by low oxygen levels known as hypoxia (52).

**Figure 3:**
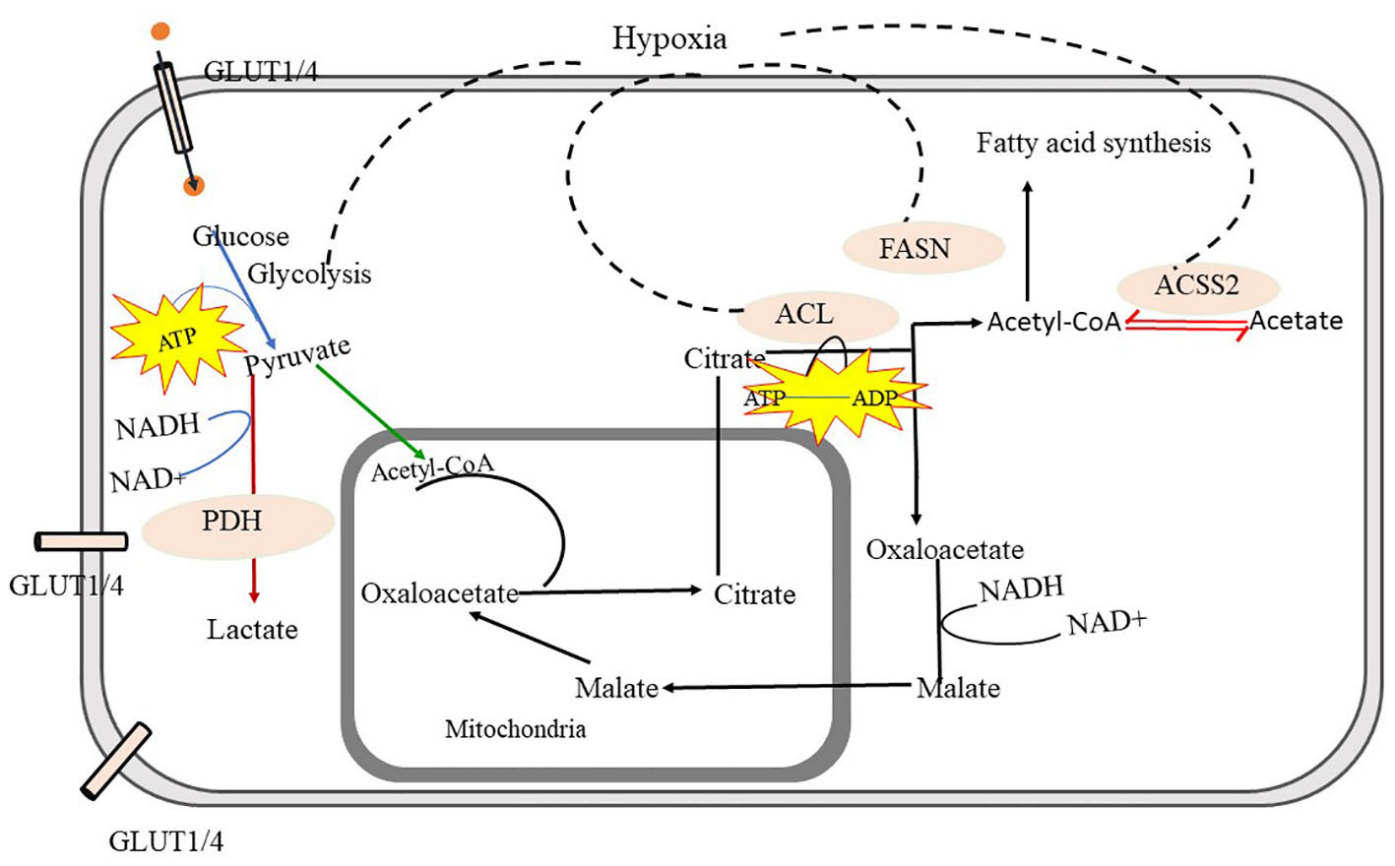
Metabolic Pathways in Malignant Form- Glycolysis is the crucial source to obtain energy through which glucose is converted into pyruvate. Pyruvate is further converted into acetyl-CoA and gets transferred into mitochondria, through the mediation of pyruvate dehydrogenase (PDH) and converted into lactate via fermentation. The tricarboxylic acid cycle (TCA) in mitochondria provides Citrate. Citrate is exported to cytosol and converted into acetyl-CoA and oxaloacetate with the help of ATP citrate lyase (ACL). Cytosolic acetyl-CoA is utilized in fatty-acid synthesis and oxaloacetate is further converted to malate and again malate is imported back into mitochondria and converted into oxaloacetate in malignant cells. cytosolic acetyl-CoA synthetase (ACSS2) facilitates the interconversion between acetyl-CoA and acetate, which helps to maintain the microenvironment in malignant cells. further fatty acid synthase (FASN) facilitates fatty acid synthesis using acetyl-CoA. In malignant cells, glycolysis, fatty acid synthesis via FACN, ACL, and ACSS, and import of oxaloacetate into mitochondria is highly increased. ATP citrate lyase -ACL, Acetyl-CoA synthetase- (ACSS2), Fatty acid synthesis (FACN).

The PI3K/Akt pathway is one of the most commonly altered pathways in human cancer. This pathway, along with mTOR, regulates the intake and use of various nutrients such as glucose, amino acids, nucleotides, and lipids. The PI3K/Akt pathway is downstream of several receptors such as G-protein coupled receptors (GPCR), receptor tyrosine kinase (RTK), integrins, and cytokine receptors. It is critical for maintaining regular cellular functions and enhancing cellular proliferation. While normal cells require external growth factors to activate these signaling pathways, malignant cells acquire activating mutations in PI3K subunits such as p110a (PI3CA) or enduring inactivating mutations in PTEN, a phosphatase that controls PI3K/Akt signaling (53, 54). MYC, a transcription factor, is connected to the PI3K/Akt/mTOR signaling pathway and is pivotal in regulating cellular nutrient utilization. It governs the expression of key metabolic genes such as *HK2* and *ENO1*, that facilitate glucose breakdown, and glucose transporters that lead glucose into cells. The stability of MYC is maintained by PI3K/Akt suppression of *FOXO* and *GSK3*, which otherwise target MYC for degradation (53, 55) **Figure 3**.

## 3 Hallmarks of T-cell malignancies

T-cell malignancy, also known as T-cell leukemia or lymphoma, represents an aggressive form of T-cell cancer characterized by the uncontrolled proliferation of T lymphocytes, a type of white blood cells responsible for immune responses. Various factors contribute to the development of T-cell malignancies, including genetic abnormalities and chromosomal rearrangements that disrupt the normal cell cycle and apoptotic mechanisms (56). These abnormalities lead to rapid and uncontrolled proliferation of malignant T-cells, enabling their survival and accumulation in the body. Glucose and glutamine serve as essential fuel sources for this aberrant proliferation (57). The tumor microenvironment may also play a role in triggering the formation of T-cell malignancies, as the presence of a small population of T-cells within the tumor microenvironment can be negatively regulated by PD-L1 (programmed cell death ligand 1) (58).

T-cell non-Hodgkin lymphomas (NHLs) are a subtype of NHLs and represent rare cancers, accounting for approximately 12% of all lymphomas (56). The prevalence of T-cell NHLs varies across different geographic regions, ranging from 18.3% in Hong Kong to 1.5% in Vancouver, British Columbia, Canada. This disparity may be attributed to varying exposure levels to pathogenic agents such as HTLV-1 and Epstein-Barr virus (EBV) in Asian countries. T-cell NHLs often manifest as extranodal diseases, making the distinction between reactive processes and lymphoma challenging due to varying rates of necrosis and apoptosis observed in biopsy specimens. However, advancements in immunophenotypic, cytogenetic, and molecular studies have significantly improved diagnostic capabilities, classification, and prognosis for T-cell NHLs (59).

T-cell malignancy is a complex and diverse disorder that is still under investigation to understand the relationship between aberrant T-cell behavior and the development of malignant cells (60). It can be categorized into T-cell lymphomas and T-cell leukemia, both of which exhibit dysfunctional T-cell characteristics. These hematologic malignancies often present with aggressive features, such as high tumor burden, elevated white blood cell counts, and the presence of large thymic masses and pleural effusions. The rapid tumor growth observed in T-cell malignancies may be attributed to the ability of immature thymocytes to undergo high rates of division during critical stages of early T-cell development. This accelerated growth is fueled by the activation of oncogenic pathways that regulate cell growth and proliferation in early T-cell progenitors (60). T-cell lymphoid malignancies exhibit heterogeneity and display thymus-specific cell surface markers. The most common T-cell cancers in children (10-15%) and young adults (25%) are T-ALL and T-LLy (61). T-ALL arises from the dysfunction of T-cells due to translocations involving specific genes associated with T-cell receptors (43). T-cell lymphoblastic leukemia, on the other hand, originates from the malignant transformation of T-cell progenitors. Adult T-cell leukemia/lymphoma (ATL) was first identified as an aggressive leukemia/lymphoma of mature T cells in Kyoto, Japan, in 1977 by Uchyama and colleagues. It was later discovered that the pathogen responsible for ATL is the human T-cell lymphotropic virus type-1 (HTLV-1), which was found in a patient with an aggressive form of mycosis fungoides three years later. HTLV-1 is endemic in the Caribbean Islands, central Africa, and certain regions of Japan, particularly Kyushu and Okinawa. Transmission of HTLV-1 occurs through breast milk from carrier mothers, sexual contact (mainly between males and females), blood transfusions, and the sharing of contaminated intravenous needles. After HTLV-1 infection, only a subset of carriers (6% male and 2% female patients) develop ATL after a long latent period. HTLV-1 is not very dominant and known in India, but there has been a recent increase in ATL cases in a tertiary healthcare center in northern Kerala. ATL can manifest in various forms, including acute, chronic, lymphomatous, and smoldering types, with skin lesions often appearing initially (44). Other forms of T-cell malignancy include T-cell acute lymphoblastic leukemia/lymphoma (T-ALL) arising from T-cell precursors, such as thymocytes, as well as T-cell large granular lymphocyte (LGL) leukemia, adult T-cell leukemia/lymphoma (ATL), and T-cell prolymphocytic leukemia (T-PLL) originating from mature T cells (62). T-cell lymphomas are rare and aggressive cancers with a poor prognosis, often due to chemotherapy resistance and patient intolerance to existing toxic chemotherapy regimens (45). T-cell lymphoblastic leukemia is considered a model for studying cancer genesis and development. Despite extensive research on the genetic rewiring of T-cell lymphoblastic leukemia, the precise molecular mechanisms underlying the transition from precursor T-cells to a malignant form remain unclear (46).

The development of T-cell malignancy involves complex interactions between environmental factors and genetics. Whole-genome sequencing studies have revealed distinct epigenetic signatures in exhausted T cells. Abnormal chromatin landscapes and altered epigenetic states have been observed in exhausted or dysfunctional T cells, closely linked to their functional state (58). Specific markers exhibit upregulation or downregulation in malignancy. Following repeated antigenic stimulation, high expression of PD-1 and other co-inhibitory molecules has been identified as an indicator of T-cell exhaustion and dysfunction, particularly in cells isolated from the tumor microenvironment (63). Epigenetic factors such as DNA methylation and histone modification control PD-1 expression and T-cell dysfunction. DNA methylation enzymes DNMT1 and DNMT3B are increased in dysfunctional or exhausted T cells, and inhibition of DNA methylation can restore normal T-cell function (64). DNA methylation plays a significant role in the expression of many genes involved in cell growth, differentiation, and genomic integrity during lineage commitment of lymphoid precursor cells, contributing to the production of mature lymphocytes with effective antigen receptors (65). Decreased expression of DNMT1 leads to gene hypomethylation, resulting in increased production of IL-2 and IFN-γ in T lymphocytes. IL-2 and IFN-γ play crucial roles in T-cell differentiation (66). Increased DNMT expression and subsequent hypermethylation of the cyclin-dependent kinase inhibitor p15INK4B have been associated with acute myeloid leukemia (AML) and the acute phase of chronic myeloid leukemia (CML) (67).

Malignant or dysfunctional T cells exhibit alterations in the expression levels of various markers. The expression of CD30 marker is increased in T-cell lymphomas. CD30 is a glycoprotein belonging to the tumor necrosis factor receptor superfamily and serves as a marker of T-cell activation and proliferation. CD30 receptor (CD30R) interaction with CD30 ligand enhances T-cell survival through the stimulation of NF-kB and MAP kinase pathways. Previous studies suggest that CD30 promotes the survival of malignant T cells by activating NF-kB and anti-apoptotic factors (68). This marker is specifically involved in the differentiation of CD4+ cells into Th2 cells and stimulates the synthesis and secretion of Th2-associated cytokines interleukin-13 (IL-13) and interleukin-4 (IL-4) while inhibiting the expression of Treg cells (69). CD30 also promotes the production of proinflammatory cytokines such as tumor necrosis factor-α (TNF-α), contributing to inflammation and the development of malignancy. Interleukin-2 receptor (IL-2R), also known as CD25, exhibits upregulation in malignant T cells. IL-2R plays a role in T-cell proliferation, activation, and survival. CD25 upregulation is associated with the expression of regulatory T cells (Treg), which play a crucial role in immunological tolerance by suppressing immune response generation and activating CD8+ cells and NK cells, thus aiding in immune escape (70, 71). Increased IL-2 production promotes T-cell proliferation (72). CD52 receptor, also known as Campath 1 antigen, is also upregulated in malignant T cells and is associated with immune tolerance (73). The upregulation of these receptors contributes to immune evasion. On the other hand, some markers show downregulation in malignant T cells. The T-cell receptor complex (TCR), an essential component of the normal T-cell immune response, consists of TCR proteins responsible for recognizing specific antigens presented by major histocompatibility complex (MHC) molecules on antigen-presenting cells (APCs). The expression of TCR complex markers is reduced or diminished in malignant T cells, leading to functional consequences. Studies suggest the presence of dysfunctional assembly of the beta chain of the TCR complex in malignant T cells (74, 75). CD3 marker expression is also decreased in malignant T cells. CD3 promotes T-cell differentiation into various T-cell subtypes, and its reduced expression level restricts T-cell differentiation (76).

### 3.1 Metabolic reprogramming in T-cell malignancy

Malignant T-cells show various alterations in terms of their metabolic reprogramming to fuel their survival and vigorous proliferation and to meet these demands rewire their several signaling pathways like Glycolysis and glutaminolysis pathways to meet the demands of energy,

#### 3.1.1 Glucose metabolism in malignant T-cells

Aerobic glycolysis or Warburg effect is the phenomenon of glucose fermentation into lactate or lactic acid in the presence of oxygen i.e., in aerobic conditions. This process provides anabolic carbons that can be used in biosynthetic pathways. Glucose uptake in normal cells is strictly regulated but, malignant cells have been reprogrammed to fuel unconstrained proliferation (77) and many malignant cell types have been reported to take up glucose at unusually elevated rates. Glucose could be transformed into a variety of cell-building components like lipids, amino acids, and, nucleotides, necessary for the development and proliferation of malignant cells. Aerobic glycolysis promotes the generation of electron carriers, which are required as cofactors in the redox process in cells. Elevated glucose uptake allows for increased synthesis of the reducing equivalent NADPH via the oxidative branch of the pentose phosphate pathways, whereas fermentation entails regeneration of the oxidizing NAD+ via the activity of lactate dehydrogenase (LDH) (78). These electron carriers are required for cellular redox balance and biosynthesis. Under hypoxic circumstances, aerobic glycolysis optimizes ATP generation. Hypoxia, or low oxygen level, is prevalent in solid tumors due to insufficient blood flow. Hypoxia can activate hypoxia-inducible factor-1α (HIF-1α), a transcription factor that controls numerous glycolysis-related genes. HIF-1α is a key regulator of cellular response to hypoxia condition and it also regulates the expression of genes that are responsible for angiogenesis, erythropoiesis, and acidosis regulation (79). HIF-1α induces genes like vascular endothelial growth factor (VEGF) (80). Angiogenesis generation of new blood vessels in developing malignant cells in an uncontrolled manner to meet the demand for oxygen and nutrients. On the contrary, it results in some disorganized structure with branching and splitting forming a chaotic and leaky network (81). Such a clumsy structure leads to inadequate blood flow, anaerobic conditions (hypoxic environment), and the generation of free radicals. Anaerobic glycolysis leads to the generation of lactate which makes the tumor environment acidic and increases the free radical load. “Earlier” lactate is considered a by-product of enhanced aerobic glycolysis and accumulated in malignant cells, raising the pH of the tumor microenvironment but a recent investigation by Brandon et al. suggests that it fuels the tricarboxylic acid cycle (TCA) (48). Lactate promotes HIF-1α activity by blocking the prolyl hydroxylases 2 (PDH 2). However, the specific mechanism is still unclear (49). When PDH 2 gets inhibited HIF-1α localized into the nucleus and binds with its other subunit HIF-1β. Further, this complex binds with hypoxia-responsive elements (HREs) and activates the gene expression for the NOX genes (NOX1, NOX 2, NOX 4, and NOX 5) NOX enzymes also contribute to the production of free radicals Superoxide and hydrogen peroxide (50) by transferring the electrons from NADPH to oxygen (51).

#### 3.1.2 Amino acid metabolism in malignant T-cells

Amino acids are one of the crucial nutrients for T cells, and they have various roles in T cell metabolism, such as building blocks for protein synthesis, sources of energy, and regulators of signaling pathways. A different subset of T-cells has a distinct metabolic profile and preferences for amino acids. Essential and non-essential amino acids also uptake get rewired in malignant T-cells. Glutamate is the essential amino acid that provides energy to a malignant form of T-cells along with enhanced glycolysis. Glutaminolysis is the conversion of glutamine an amino acid, into glutamate and then into α-ketoglutrate, which can enter the TCA cycle to generate energy and biosynthetic substrate. It fuels the TCA cycles and supports anabolic processes, such as nucleotide synthesis. It also modulates the redox balance and the epigenetic regulation of T-cells by producing NADPH and donating methyl group. Glutaminolysis pathways regulate the mTOR pathway to control T-cell proliferation (82). Various factors impact the survival and proliferation of malignant T-cells like TEM which provide stimulation via cytokines environment, antigen stimulation environments, and crucial factors.

Glutamines (Glu) are transported into T-cells via amino acid transporters (AATs), which are sodium (Na+)-dependent transporters. These transporters use concentration gradients to allow Glu and Na+ ions into the cell and expel any excess concentration of Na+ ions. The transporters that perform this function belong to the solute carrier protein 1 member 5 (*SLC1A5*) and *SLC38A*1 families, with *ASCT*2 being the most important. Subsequently, Glutamine (Glu) enters the mitochondria via the *SLC1A*5 variant (SLC1A5_var), which is an AAT located on the mitochondrial membrane through its N-terminal targeting signal. Here, Gln is decomposed into glutamate (Glu) and ammonia by the action of mitochondrial glutaminase. Alternatively, Gln can be transformed into α-ketoglutaric acid via glutamate dehydrogenase 1 (GLUD1) (83).

Arginine metabolism is the conversion of arginine to ornithine and urea using arginase, or into citrulline and nitric oxide (NO) through nitric oxide synthase (NOS). Arginine metabolism is crucial for nucleotide synthesis, NO production, urea cycle, and immune regulation. Different subsets of T-cells, and other immune cells in the tumor microenvironment modulating arginine metabolism.

In summary, metabolic reprogramming in cancer cells, including T-cell malignancies, is associated with altered nutrient utilization and adaptations to the TME. Understanding the specific metabolic pathways involved in T-cell malignancies can pave the way for developing targeted therapies. The PI3K/Akt/mTOR pathway is among the key metabolic features observed in malignant T cells.

## 4 Melatonin

**Figure 4:**
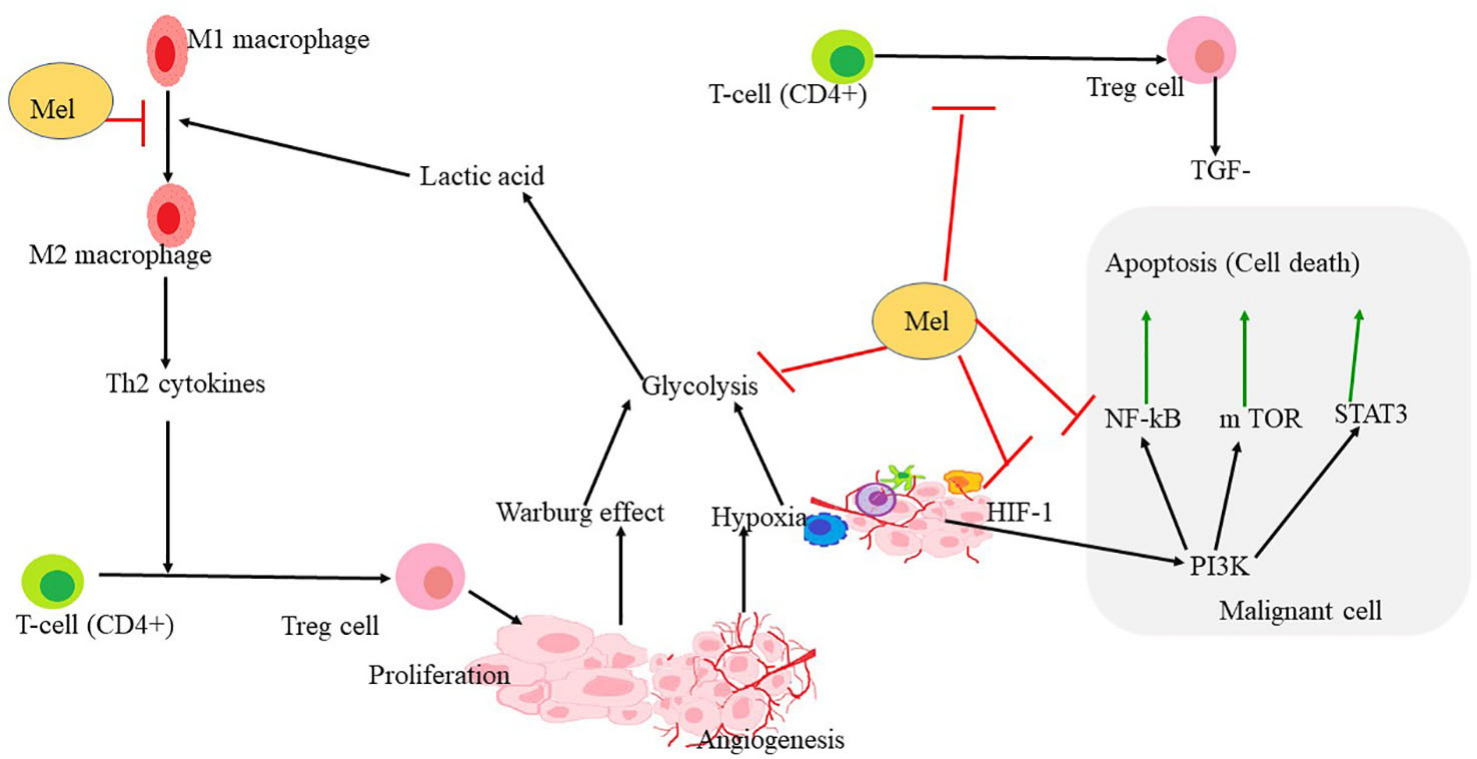
Mechanism through which melatonin rewires the pathways involved in hypoxia and angiogenesis formation. HIF1α is the key regulator of hypoxia and angiogenesis. Polarization of macrophage to M2 also triggers the proliferation of Treg-cells, leading to the upregulation of some apoptotic factors in malignant cells. PI3K, NF- κB, STAT3 leading to apoptosis that may be upregulated in hypoxia conditions or in the tumor microenvironment. Melatonin reverses the polarization of macrophages and inhibits Treg-cell proliferation and angiogenesis. Melatonin further inhibits HIF-1α, NF-κ B, and also STAT 3 and PI3K pathways leading to inhibition of glycolysis, ultimately leading to apoptosis.

Melatonin (N-acetyl-5-methoxytryptamine), is a naturally occurring indole amine produced by the human body through various mechanisms. Its primary production occurs in the pineal gland, particularly in response to darkness. In addition to the pineal gland, other organs such as the gastrointestinal tract, bone marrow, lymphocytes, retina, and skin are known to synthesize melatonin (84). The suprachiasmatic nucleus (SCN) of the hypothalamus serves as the biological clock governing the 24-hour rhythm of melatonin production and secretion (85). Melatonin levels rise during the night and tend to rise during the day and night, gradually declining in the morning and throughout the day. Elevated melatonin levels at night play a vital role in orchestrating optimal homeostatic metabolic rhythms in target organs, contributing to the prevention of numerous diseases. Exposure to the light at night can disrupt the circadian rhythm and hamper melatonin generation. Melatonin is a pleiotropic hormone with diverse functions, including anti-oxidants, oncostatic, antiaging, and immune modulation activities (86–88). It functions by inhibiting the production of pro-oxidants and promoting the synthesis of antioxidants such as glutathione peroxidase and superoxide dismutase, thereby offering protection against reactive oxygen and nitrogen species (ROS/RNS) (89). Additionally, melatonin has demonstrated cardioprotective effects. Due to its lipophilic nature, melatonin can easily traverse various morphological and physiological barriers and reach the subcellular compartments of heart cells, mitigating oxidative stress generation (90). This productive action helps prevent DNA damage, which is essential for maintaining genomic stability (86, 90, 91). It exerts its effects through two receptors, namely MT1R and MT2R, which belong to the family of G-protein coupled receptors (GPCRs) and regulate various physiological processes (88) (**Figure 4**).

### 4.1 Anti-oncogenic/oncostatic impact of melatonin on various cancer cells

The concentration of melatonin in cerebrospinal fluid (CSF) and blood reaches its peak between 2:00 a.m. and 4:00 a.m. and then gradually decreases as the day progresses. During night time, the melatonin concentration in the blood typically ranges from 80 to 120 pg/ml, but it goes down to 10-20pg/ml during the daytime (92). The melatonin concentration is usually deficient, typically ranging from 0.1 to 0.6 pg/ml. This is significantly lower than the standard level of melatonin found in healthy humans, which can reach up to 120 pg/ml at night. This indicates that cancer cells may have difficulties in producing melatonin or maybe breaking it down more quickly, which could be contributing to their malignant behavior (**Table 1**).

**Table 1:** Melatonin levels in some malignant cell lines.

Melatonin concentration	Cancer cells	Inference	References
0.1 pg/ml	Breast cancer cells (MCF-7)	Melatonin helps docetaxel, a cancer drug work better and safer on breast cancer cells. *In vitro* studies suggest melatonin can improve breast cancer therapy.	(93)
0.6 pg/ml	Human glioma cell (U87MG)	Melatonin could be a potent therapeutic drug for glioblastoma by inducing apoptosis via the Akt/mTOR pathway.	(94)

Numerous in vivo and in vitro studies have demonstrated that melatonin can suppress cancer development. It interacts with several metabolic pathways and plays a modulatory role in estrogen-controlled pathways in breast cancer, potentially exerting an oncostatic effect (95, 96). Different pharmaceutical doses of melatonin are given by Meharzadi et al. (13) (**Table 2**).

**Table 2:** Different pharmaceutical doses of melatonin.

Cancer cells	Melatonin dose	Inferences	Model	References
Lung cancer	1 nm, 1μg, 1mm	Enhances immunomodulatory impacts.	*In-vitro* SK-LU-1	(97)
Leukemia	1 mM	Protect normal cells from ROS.	*In-vitro* HL-60	(98)
Breast cancer	2.5mg/kg	Enhance antioxidant activity	female Sprague Dawley rats	(99)
Pancreatic cancer	53.76 mg	Decrease tumor nodules and oxidative stress and death.	Male Syrian hamster	(100)
Hepatocellular carcinoma	1-100 μM	Protected healthy cells from chemotherapy-mediated ROS production.	HCC24/KMUH	(101)
Cervical cancer	10-1000μg	Melatonin has dose-dependent activity	HeLa	(102)
Hepatocellular carcinoma	20μg	Inhibit oxidative stress and induce apoptosis.	Adult female rats	(103)
Bladder cancer	1mM 1000mg/kg	Inhibit angiogenesis and metastasis.	HT1197,HT1376,T24,male C57B/L6 mice	(104)
Lung cancer	0.25-2.5mM	Induce apoptosis.	A549,H1299	(105)
Neuroblastoma	10μg	Reduce the neurotoxicity.	SH-LU-1	(106)
Breast cancer	0.3Mm	Enhance doxorubicin effects by apoptosis and cancer cell death	MCF-7	(107)

### 4.2 Action mechanism of melatonin in cancer cell

Melatonin exhibits the ability to attach to distinct types of receptors, situated both along the cell membrane and within the cellular nucleus. The membrane receptors are MT1R and MT2R, a type of G-Protein coupled receptor (GPCR). These receptors are prevalent in humans and other mammals, affecting diverse processes like circadian rhythm, immune modulation, and blood pressure regulation (22). Within the cell nucleus, the binding locations referred to as orphan receptor retinoic-acid receptor (ROR) ROR-α and orphan receptor retinoid Z receptor (RZR) RZR-β orphan receptors engaged in gene regulation. Some studies suggest that these receptors regulate the gene expression which is involved in inflammation, metabolism, and bone formation (21). Furthermore, melatonin can also attach to other membranes with the ROR family, including RZR-β AND ROR-γ. Previous reports suggest that human CD4+ cells which are helper T-cells and CD8+ cells which are cytotoxic T-cells have a higher affinity for melatonin and may respond to blood serum melatonin levels 150-200pg/mL, as they express both membrane receptors MTIR and nuclear receptors RZR-α, ROR-α_1_, ROR-α_2,_ for melatonin. T-cells also express the enzymes which are crucial for the biosynthesis of melatonin like tryptophan-5-hydroxylase (TPH), L-aromatic amino acid decarboxylase (AADC), N-acetyltransferase (NAT), and Hydroxyindole-O-methyltransferase (HIOMT) (25). This suggests that melatonin has an important role in regulating immune cells as other Peripheral blood mononuclear cells PBMCs also express these receptors and enzymes giving a strong viewpoint on melatonin as an immune modulation and impacting the T-cell functions. Melatonin also influences the survival of T-memory cells (25). Most active T-cells undergo apoptosis but a few subsets of memory T-cells remain alive. T memory cells can recall and react to previous encounters with pathogens. These memory T-cells (Tm) are sub-categorized into effector T-cell (Tem), memory stem T-cell (Tscm), Follicular helper T-cells, and resident memory T-cells (Trm). Melatonin declines the apoptosis of human and mouse T-cells via TCR/CD3 and suppresses the CD3-driven expression of CD95L. CD95L is a protein that belongs to the Tumor necrosis factor (TNF) family. It can bind with the CD95 marker expressed on T-lymphocytes and other cells. CD95L triggers several pathways leading to cell death or survival depending on the context. Inhibiting the CD95L expression increases the survival of Tm cells by preventing apoptosis (108). Melatonin influences the production of IL-2 a cytokine crucial for immune response. Melatonin stimulates the Th1 cells to produce IL-2 and CGP 52608 which operates similarly to melatonin (a ligand for RZR/ROR) (109). Whereas in Jurkat cells CGP52608 an antagonist to the melatonin nuclear receptor inhibits the secretion and production of IL-2. Suggests that melatonin modulates the T-cells via nuclear receptors (110). Th17 cells, a category of T-lymphocytes that generate IL-17, cytokines causing inflammation and immune harm, are influenced by melatonin (111). Melatonin restrains the development of Th17 cells, a characteristic that could prove beneficial for autoimmune conditions. Melatonin stimulates ROR-α to translocate from the nucleus to the cytoplasm where it loses the capacity to activate its target genes. Consequently, this decreases the expression of RORγt another nuclear receptor essential for TH17 development. Melatonin binding with MT1R a GPCR, sets off a signaling process involving ERK1/2, C/EBPα, REV-ERBα, and NFIL3. These proteins control the expression of genes related to Th17 maturation by binding to their promotor regions. Melatonin’s action extended to decrease the expression of RORα and RORγt, achieved by inhibiting REV-ERB-α and NFIL3, respectively. Binding with MT1R phosphorylation of Erk1/2 occurs which further stimulates CAAT/enhancer binding protein α (C/EBPAα) which suppresses the REV-ERBα (expressed by nr1d1 gene) which further suppresses the expression of NFIL3 (transcription factor inhibits the RORα and RORγt (112, 113).

Malignancy is the abnormal development and proliferation of malignant cells, which is associated with various pathways such as the PI3K/Akt/mTOR pathways, insulin-like growth factor-1 (IGF-1), hypoxia-inducible factor-1 (HIF-1), and NF-KB. These pathways play a direct role in promoting tumor development in the tumor microenvironment (TME), a condition characterized by low oxygen levels known as hypoxia (52). Melatonin, a hormone produced by the pineal gland, regulates a variety of physiological activities. Numerous studies have demonstrated its significant impact on various cancer cell lines and their programmed cell death, also known as apoptosis. However, the effects of melatonin may vary depending on specific carcinogenic molecules and the growth medium conditions (114, 115).

Melatonin inhibits tumor growth by suppressing the actions of prolactin-insulin-like growth factor-1 (IGF-1), growth hormone-dependent growth factors (GHFs), epidermal growth factor receptor (EGFR), vascular endothelial growth factor (VEGF), hepatocyte growth factor (HGF), transforming growth factor (TGF), and platelet-derived growth factors (PDGF). By blocking these growth factors, melatonin indirectly hampers the malignant transformation of healthy cells (32). Moreover, melatonin induces mitochondrial-dependent activities and promotes apoptosis in tumor cells. Cancer cells in the tumor microenvironment undergo altered metabolic rewiring, converting glucose to lactate even in the presence of oxygen. Lactate creates an acidic environment that favors cell proliferation, but melatonin counteracts this effect by inhibiting the mitochondrial enzyme pyruvate dehydrogenase kinase (PDK) and stimulating the production of acetyl-CoA in the tumor microenvironment (TME) (116).

### 4.3 Melatonin influencing the metabolic rewiring in T-cell malignancy

Metabolic rewiring is a powerful strategy employed by malignant cells to adapt to different environmental conditions and physiological demands. Through metabolic rewiring, the metabolic pathways are modified and preferences, ultimately influencing crucial cellular functions such as energy production, signaling mechanism, biosynthesis, and survival (117). Metabolic reprogramming has been implicated in cancer progression (118). Malignant cell metabolism differs significantly from that of normal cells to meet the biosynthetic and bioenergetic demands of rapid proliferation and adaptation to the tumor microenvironment (TME) (119). Cancer cell metabolic reprogramming and adaptability play crucial roles in therapy resistance. The characteristics and activity of immune cells within the TME are closely associated with cancer growth, and the metabolic activity of cancer cells influences the TME (120). This metabolic interplay alters nutrient availability in the microenvironment as cancer cells and the immune system compete for essential nutrients like glucose, glutamine, lipids, and amino acids (121).

**Figure 5:**
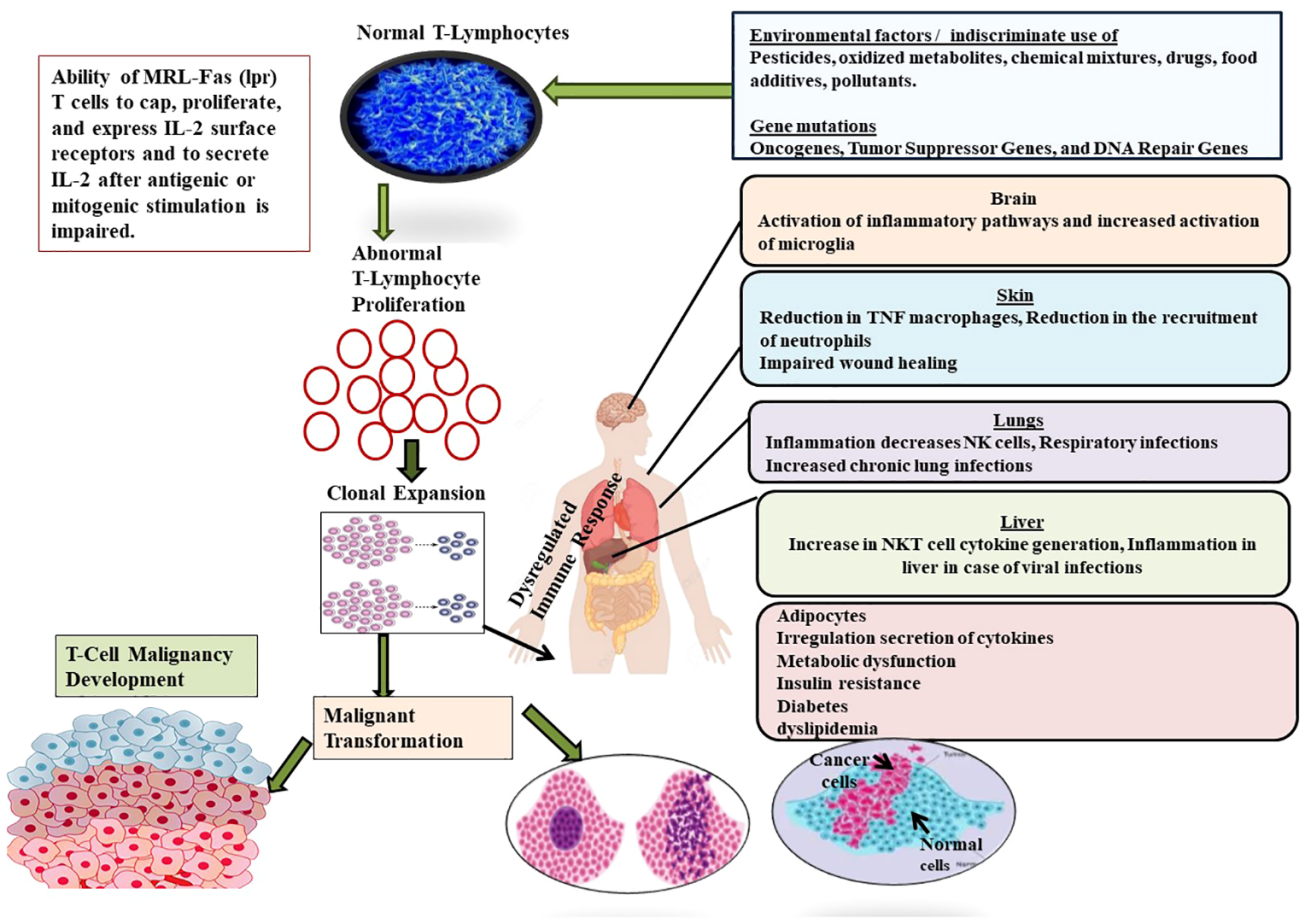
Showing the role of different factors such as environmental factors (Pesticides, oxidized metabolites, chemical mixtures, drugs, food additives, pollutants) and genetic factors (Oncogenes, Tumor Suppressor Genes, and DNA Repair Genes) in the development of T-cell malignancy. Malignancy in T-cell induces complications in different organs such as brain, liver, skin, lungs and finally suppresses the formation and development of normal T-cells.

T-cell malignancies are characterized by dysregulated metabolic pathways that sustain the increased energy and metabolic demands of malignant cells Identifying these metabolic pathways is crucial for developing novel therapeutics targeting the metabolic vulnerabilities of T-cell malignancies (43). The development and progression of T-cell malignancies rely on various pathways involved in metabolic regulation (120). Tumor cells exhibit enhanced glycolysis rates even in the presence of adequate oxygen, a phenomenon known as the Warburg effect (122). This metabolic shift enables cancer cells to generate the energy and biosynthetic precursors necessary for growth and survival. Malignant T-cells undergo a significant metabolic shift. This entails an increased dependency on glycolysis, and glutaminolysis as well as a modification in lipid metabolism. The Warburg effect describes cancer cells’ preference for anaerobic glycolysis over aerobic glycolysis, even under normal aerobic conditions (Warburg, 1956). Anaerobic glycolysis facilitates rapid ATP production (123). All these metabolic reprogramming increases the free radical loads. Nicotinamide adenine dinucleotide (NAD) plays a vital role in redox reactions within various metabolic pathways, DNA repair, and post-translational modifications (124). Malignant cells require elevated levels of NAD for their survival and proliferation (124). Nicotinamide adenine dinucleotide phosphate (NADPH) protects tumor cells from reactive oxygen species (ROS) and reactive nitrogen species (RNS). Maintaining a balanced level of ROS is necessary for cancer cells’ signal transduction, but excessive ROS can trigger cytotoxicity, DNA damage, and cellular apoptosis. To overcome this, tumor cells develop a defense system that regulates the level of antioxidants required for their survival, and this defense system depends on NADPH production (125). Lipid metabolism rewiring is the major aspect of metabolic rewiring in malignant T-cells. Lipids are present in the form of droplets, and rafts, act as signaling molecules and as a precursor for the synthesis of macromolecules. Lipid rafts activate the expression of fatty acid synthase (FASN) in malignant cells (126). FASN is an enzyme that catalyzes fatty acid synthesis through acetyl-CoA. Fatty acids are utilized by mitochondria to generate ATP (117) (**Figure 5**).

Previous research has demonstrated that melatonin directly influences T/B cell activation. During T-cell activation, the expression of CD38 and CD69 on the cell surface is often observed. CD38 is an NAD+ glycosyl hydrolase/ADP ribose cyclase involved in cell adhesion and signaling. It is present in early activated T cells as well as various immunological cells, including CD4+ T cells. CD38 expression increases during infections. Additionally, CD69 serves as a well-known marker of early lymphocyte and T cell activation, primarily expressed by CD8+, and CD45RO+ activated T cells (**Table 3**).

**Table 3:** Receptors/enzymes reported in T-cells.

Name of receptor/enzyme	Author’s	Reference
Melatonin membrane receptor-1 (MT1R)	Pozo et al., 1997 Guerrero et al., 2000; Lardone et al., 2006; Pozo et al., 2004	(111, 127, 128)
Melatonin membrane receptor-2 (MT2R)	Lardone et al., 2006	(111)
Retinoic acid-related orphan receptor (ROR)	Guerrero et al., 2000; Lardone et al., 2006; Pozo et al., 2004.	(111, 128)
Retinoid Z receptor (RZR)	Guerrero et al., 2000; Lardone et al., 2006; Pozo et al., 2004.	(111, 128, 129)
N-acetylserotonin-O-methyltransferase (ASMT)	Lardone et al., 2006; Pozo et al., 2004	(111, 129)
Arylalkylamine N-acetyltransferase (AA-NAT)	Lardone et al., 2006;	(111)
Tryptophan-5-hydroxylase (TPH)	Chen et al., 2015.	(130)
L-aromatic amino acid decarboxylase (AADC)	Chen et al., 2015.	(130)

#### 4.3.1 Melatonin inhibiting glycolysis in T-cell malignancy

Glycolysis is a metabolic mechanism that transforms glucose into pyruvate and generates ATP, the primary source of energy for cells. Even in the presence of oxygen, cancer cells frequently rely on glycolysis more than normal cells. The Warburg effect is a phenomenon that permits malignant cells to survive and proliferate in hypoxia and nutrient-depleted environments. Glycolysis also provides cancer cells with biosynthetic intermediates while decreasing oxidative stress by redirecting glucose away from mitochondrial oxidative phosphorylation (OXPHOS). One of the mechanisms by which melatonin exerts its anti-cancer effect is by inhibiting glycolysis (13).

##### 4.3.1.1 Melatonin inhibiting the Hexokinase II

Hexokinase II plays a pivotal role in the glycolytic pathway, which is a series of chemical reactions that convert glucose into energy in the form of adenosine triphosphate (ATP). In the first step of glycolysis, HKII phosphorylates glucose, which is essential for trapping glucose inside the cell and preparing it for further processing in the glycolytic pathway. In other words, HKII acts as a gatekeeper, ensuring that glucose is efficiently utilized for energy production within the cells (131, 132).

Melatonin has been proven to suppress the action of HKII by binding to the enzyme directly, modifying its structure and activity. This interaction stops HKII from phosphorylating glucose and inhibiting glycolysis efficiently. Because of this glucose trapping is reduced and glycolysis cannot continue as normal leading to a decrease in the level of glycolysis and hence the energy supply to malignant cells declines (133).

##### 4.3.1.2 Melatonin activating AMP-activated protein kinase and reducing the Glut1 expression

Melatonin stimulates the enzyme AMP-activated protein kinase (AMPPK). AMPK is an important cellular energy sensor that detects variations in the Adenosine monophosphate (AMP) to adenosine triphosphate (ATP) ratio. AMPK is activated when cellular energy levels are low and the AMP/ATP ratio rises (indicating an energy deficit). This activation has numerous consequences for glycolysis inhibition (134). AMPK controls the mTOR signaling pathway. mTOR is a key regulator of cell growth and metabolism. It combines signals from many sources, including growth factors, nutrient availability, and energy status. Melatonin activation of AMPK inhibits mTOR signaling (135).

Glut 1 expression is reduced when is inhibited by AMPK. Glut1 is a protein that aids in the transport of glucose into cells. Melatonin indirectly decreases the amount of glucose available for glycolysis by lowering the expression of Glut1 (134, 136).

### 4.4 Melatonin inducing apoptotic proteins in malignant T-cells

Metabolic reprogramming is necessary for T-cell activation and differentiation to fulfill new demands, including increased synthesis of various inflammatory mediators. Upon T-cell receptor (TCR) activation, there is an upregulation of glucose, amino acid, and iron uptake transporters (137). These transporters are essential for the differentiation of effector T cells. Consequently, the nutritional microenvironment surrounding T cells has the potential to modulate T-cell immunological responses (137). Melatonin induces apoptosis of malignant cells through intrinsic and extrinsic pathways. The intrinsic pathways of apoptosis are activated by melatonin by impacting the membrane potential of mitochondria and cytochrome c release via increasing the expression of pro-apoptotic genes belonging to Bcl-2 family like Bax, Bak, Bid these proteins from pores in the mitochondrial membrane and facilitating the release of cytochrome c (138). Cytochrome c is released from the mitochondrial permeability transition pore (MPTP) due to damage or stress in mitochondria (138). Now further cytochrome c activates other proteolytic activity via caspases. Caspase activity leads to apoptosis (138).

### 4.5 Melatonin and p53 targeting malignant T-cells

The p53 encoded by the *TP53* gene, a tumor suppressor protein, regulates the cell death/cell cycle arrest and acts as a transcription factor. DNA damage in cells is sensed by p53 leading to cell cycle arrest or cell death (139). The role of p53 in tumor suppression has been identified as an intricate combination of several biological mechanisms and rewiring the metabolic pathways thereby, suppressing the tumor progression (140). Under these, the control of cellular metabolism and autophagy, as well as inhibiting the ferroptosis process, have a substantial influence on how well cells can respond and adapt to disturbance, limiting the build-up of damage that can lead to cancer. By restricting glycolysis, p53 promotes mitochondrial respiration. The glucose transporters GLUT1, GLUT3, and GLUT4 are repressed (141). Furthermore, p53 inhibits the expression of TIGAR (TP53-inducible glucolysis and apoptosis regulator), a regulator of glucose breakdown. TIGAR expression decreases the fructose-2,6 bisphosphate expression in cells inhibiting glycolysis (142) as well as PDK2 (pyruvate dehydrogenase kinase 2), which inactivates the pyruvate dehydrogenase complex controlling pyruvate access to the Kreb cycle (131).

Melatonin inhibits the STAT 3 signaling which enhances the p53 expression. p53 also regulates the immune system. It controls the inflammatory response via STAT3, which is triggered by the inflammatory cytokine IL-6 (143) In studies, it was reported that p53 population declines in pancreatic cancer lead to an increase in STAT3 activation. STAT3 is proinflammatory and makes the environment suitable for the progression of tumors (144).

## 5 Summary and conclusion

The emerging evidence highlights melatonin as a significant modulator in the metabolic rewiring processes observed in T-cell malignancies through its pleiotropic effects. on various cellular pathways and molecular targets, melatonin exerts regulatory control over key metabolic alterations seen in these malignancies. It may be suggested that melatonin by promoting mitochondrial-dependent activities and stimulating the production of acetyl-CoA, participates in the suppression of tumor growth and proliferation and hence disrupts the metabolic rewiring. Moreover, melatonin’s influence on nutrient uptake and metabolism in T cells, including the modulation of glucose and amino acid transporters, highlights its ability to shape the metabolic profile of malignant T cells. In addition, such modulation has implications for T-cell differentiation, function, and overall immunological responses in T-cell malignancies. The regulation of critical signaling molecules involved in T-cell activation, such as CD38 and CD69, further underscores melatonin’s role in modulating T-cell behavior and immune responses within the tumor microenvironment.

Understandings of the research evidence present the picture more clearly that harnessing the therapeutic potential of melatonin in T-cell malignancies may open new avenues for targeted interventions by exploiting its effects on metabolic rewiring and T-cell activation. Therefore, melatonin-based strategies may be offered as novel therapeutic approaches to combat these aggressive hematological cancers. However, further research is needed to unravel the precise molecular mechanisms underlying melatonin’s modulatory effects on metabolic rewiring in T-cell malignancies and to establish an effective pharmacological dose. Additionally, well-designed clinical studies are warranted to validate its efficacy and safety as an adjunctive therapy or standalone treatment option to disrupt the metabolic rewiring processes driving T-cell malignancies, thereby offering potential improvements in patient outcomes and survival rates.

## 6 Recommendations

### 6.1 Mechanistic studies

Perform extensive mechanistic studies to uncover the precise signaling pathways and molecular targets involved in melatonin’s impact on metabolic reprogramming in T-cell cancers. This will offer a valuable understanding of the underlying mechanisms and aid in pinpointing prospective therapeutic targets.

### 6.2 *In vivo* models

To comprehensively examine the effects of melatonin on tumor metabolism in a complex microenvironment, it is imperative to employ *in vivo* models such as mouse models of T-cell malignancies. This will facilitate a more physiologically relevant setting to verify the observations made *in vitro* and further investigate the impact of melatonin on metabolic rewiring.

### 6.3 Combination therapies

It is recommended to explore the potential synergies between melatonin and current treatment methods such as chemotherapy or immunotherapy. Preclinical investigations and clinical trials should be conducted to evaluate the advantages of combining melatonin with conventional therapies. The ultimate goal is to improve treatment effectiveness and overcome resistance mechanisms in T-cell cancers.

### 6.4 Immunological responses

Explore the immunomodulatory effects of melatonin in the context of T-cell malignancies. Investigate its impact on T-cell differentiation, activation, and anti-tumor immune responses. This will help elucidate the role of melatonin in shaping the immune microenvironment and its potential for combination with immunotherapies.

### 6.5 Clinical trials

Design and conduct well-controlled clinical trials to evaluate the efficacy and safety of melatonin-based interventions in patients with T-cell malignancies. Consider patient selection criteria, optimal dosing regimens, and combination strategies to determine the clinical utility of melatonin in improving patient outcomes.

### 6.6 Biomarker development

Identify and validate biomarkers associated with melatonin response in T-cell malignancies. Establish robust biomarker panels to predict patient response to melatonin-based therapies and monitor treatment effectiveness.

## 7 Future perspective

The future perspectives of this review article involve unraveling the underlying mechanisms, exploring synergistic effects with existing therapies, investigating melatonin as an adjuvant to immunotherapies, developing targeted analogs, and conducting rigorous clinical trials. By advancing our understanding and harnessing the therapeutic potential of melatonin, we can pave the way for innovative approaches to tackle metabolic rewiring in T-cell malignancies and improve patient outcomes in the future.

## Author contributions

Corresponding and joint first authors from Guru Ghasidas Vishwavidyalaya wrote and edited the manuscript. All other co-authors contributed intellectually to the designing, editing, and proofreading of the manuscript.

## Glossary

**Table:**

HSCs	Hematopoietic stem cells
HTLV-1	human T-cell leukemia virus 1
T-ALL	acute lymphoblastic leukemia
T-LLy	T-lymphoblastic lymphoma
ATLL	adult T cell leukemia/lymphoma
T-LGL	T cell large granular lymphocytic leukemia
T-PLL	T prolymphocytic leukemia
CTCL	T cell lymphomas are cutaneous T cell lymphoma
PTCL	peripheral T cell lymphoma
MF	mycosis fungoids
SS	Sezary syndrome
AITL	Angioimmunoblastic T cell lymphoma
ENKTL	extranodal natural killer (NK)-T cell lymphoma
EATL	enteropathy-associated T cell lymphoma
HSTCL	hepatosplenic T cell lymphoma
ROS	reactive oxygen species
RNS	reactive nitrogen species
VEGF	vascular endothelial growth factor
HIF-1α	hypoxia-inducible factor-1α
HER-2	human epidermal growth factor receptor 2
TNF-α	tumor necrosis factor-alpha
IL	interleukin
IL-1β	interleukin-1β
TEM	tumor microenvironment
EMC	extracellular matrix
EMT	epithelial-mesenchymal transition
LDH	lactate dehydrogenase
NADPH	nicotinamide adenine dinucleotide phosphate hydrogen
NAD+	nicotinamide adenine dinucleotide
PD-L1	programmed cell death ligand-1
VEGF	vascular endothelial growth factor
IGF	insulin-like growth factor-1
GPCR	G-protein coupled receptors
RTK	receptor tyrosine kinase
TCA	tricarboxylic acid cycle
Glu	Glutamines
GLUD1	glutamate dehydrogenase 1
NO	nitric oxide
NOS	nitric oxide synthase
MPTP	mitochondrial permeability transition pore
TIGAR	TP53-inducible glycolysis and apoptosis regulator
SCN	suprachiasmatic nucleus
ROR	orphan receptor retinoic-acid receptor
RZR	orphan receptor retinoid Z receptor
TGF	transforming growth factor
FASN	fatty acid synthase
PDGF	platelet-derived growth factors
PDK	pyruvate dehydrogenase kinase
OXPHOS	oxidative phosphorylation
HKII	Hexokinase II.
